# A Prebiotic Ribosylation of Pyrimidine Nucleobases Enabled by Metal Cations and Clay Minerals

**DOI:** 10.3390/life11121381

**Published:** 2021-12-10

**Authors:** Qian-Qian Chen, Ze-Run Zhao, Xiao Wang

**Affiliations:** School of Chemistry and Chemical Engineering, Nanjing University, 163 Xianlin Avenue, Nanjing 210023, China; MF1924003@smail.nju.edu.cn (Q.-Q.C.); DG20240153@smail.nju.edu.cn (Z.-R.Z.)

**Keywords:** origins of life, RNA world, uracil, ribosylation, metal cation, clay mineral

## Abstract

We report a prebiotically relevant solution to the *N*1-ribosylation of pyrimidine nucleobases, a well-known challenge to the RNA world hypothesis. We found that the presence of metal cations and clay minerals enable the previously unachievable direct ribosylation of uracil. Spectroscopy and chromatography analyses confirmed the formation of ribosylated uracil. The method can be extended to the ribosylation of 2-pyrimidinone. These findings are also compatible with the metal-doped-clay model, developed by our lab for the unified route of the selection of ribose and subsequent syntheses of nucleotide and RNA.

## 1. Introduction

The RNA world is one of the leading hypotheses for the chemical origin of life [1]. The construction of the glycosidic bond between the ribose and nucleobase moieties is considered the most debatable step toward the synthesis of RNA, noted for its low yield and selectivity. This “nucleosidation problem” has remained unsolved for decades [2,3,4]. The weak nucleophilicity of *N*9 of purine, and *N*1 of pyrimidine, is the major obstacle for the direct ribosylation of nucleobase [5,6]. Various conditions have been investigated since the pioneering work by Orgel in the 1970s [7,8]; compared with the *N*9 of purine, the nucleophilicity of the *N*1 of pyrimidine is even weaker. The ribosylation of uracil and cytosine has been a key stumbling block for completing the hypothetical synthesis for the RNA world [9]. There have been very few precedents on the formation of uridine or its isomers via the direct ribosylation of uracil. Among the few published examples, Hud was able to theoretically demonstrate that divalent metal ions, such as Mg^2+^, might be promotive to the ribosylation of uracil. Cations are believed to lower activation energy by holding the reactants in close proximity [10]. However, no detectable uridine, or its isomers, were reported experimentally. More recently, Zare reported the ribosylation of uracil, facilitated by generating aqueous microdroplets of the reaction solution [11,12]. Although no further evidence was given to support the formation of uracil-1-β-d-ribofuranoside (β-uridine), the reported high-resolution mass spectroscopy (HRMS) signal indicated that the ribosylation of uracil had taken place, no matter which isomer(s) was formed. The generality of the corresponding geochemical conditions of this model on the primordial Earth remains unknown. To circumvent the low reactivity of pyrimidine, a stepwise strategy was developed by Orgel [13]. In addition, Sutherland developed a stepwise route to access pyrimidine nucleotides [14]. Carell reported a multistep synthesis based on the condensation between ribose and *N*-isoxazolyl-urea, to form pyrimidine nucleotides [15]. Saladino and Di Mauro reported the detection of the ribosylated uracil catalyzed by meteorite, but without indicating the yield and the isomeric form [16]. Moreover, the prebiotic availability of the required proton irradiation is to be confirmed. Nevertheless, the direct nucleosidation between ribose and nucleobase remains attractive due to its simplicity, although a prebiotically general condition is yet to be developed.

Recently, our lab has demonstrated that metal-doped-clay (MDC) is able to highly selectively adsorb and stabilize ribose from the complex Formose mixture, as well as promote downstream syntheses toward RNA, such as nucleosidation and phosphorylation [17]. We have successfully carried out the ribosylation of formamidopyrimidine (FaPy) and melamine in the presence of MDC, with both of the nucleobases being non-canonical. It was shown that MDC-retained ribose is highly reactive in these ribosylation reactions. These results encouraged us to explore if MDC has a facilitating effect on previously unachievable transformations. In particular, it would be interesting to explore whether the more challenging canonical nucleobase can be ribosylated under similar conditions. Herein, we present the direct ribosylation of pyrimidine nucleobases, facilitated by the use of metal salts and clay minerals.

## 2. Results

**Figure 1:**
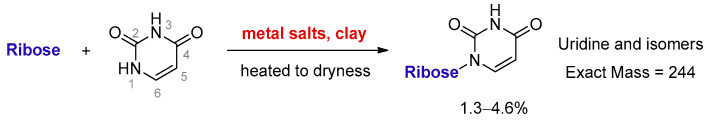
Direct ribosylation of uracil enabled by metal salts and kaolinite.

Clay minerals are an important catalyst and platform for synthesizing RNA oligomers [18]. Cation-exchanged clays have been reported to catalyse organic transformations, such as the formation of acetal [19], which is a condensation between aldehyde and alcohol. Therefore, it is reasonable to assume that MDC is able to catalyze nucleosidation, which can also be regarded as a condensation between an aldehyde (sugar) and a nucleophile (pyrimidine). Here, we discovered that uracil and ribose could couple to form a set of desirable products in the presence of metal salts and kaolinite (Figure 1).

**Figure 2:**
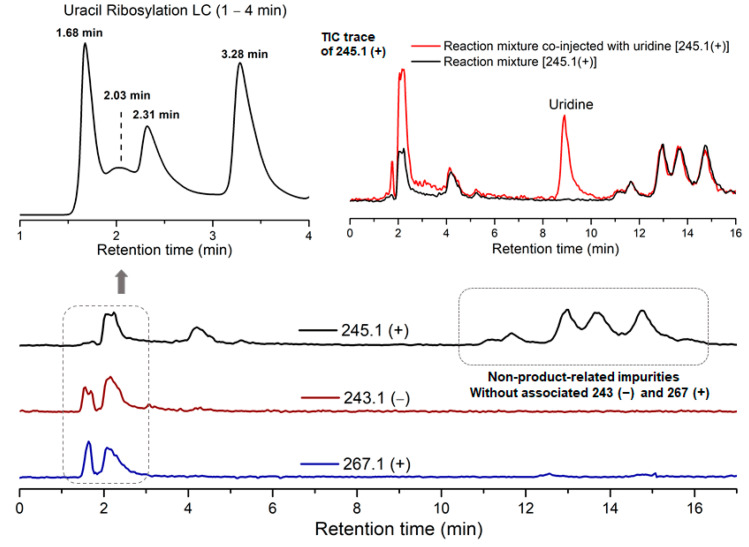
HPLC trace and TIC analysis of the characteristic masses of the ribosylation products.

**Figure 3:**
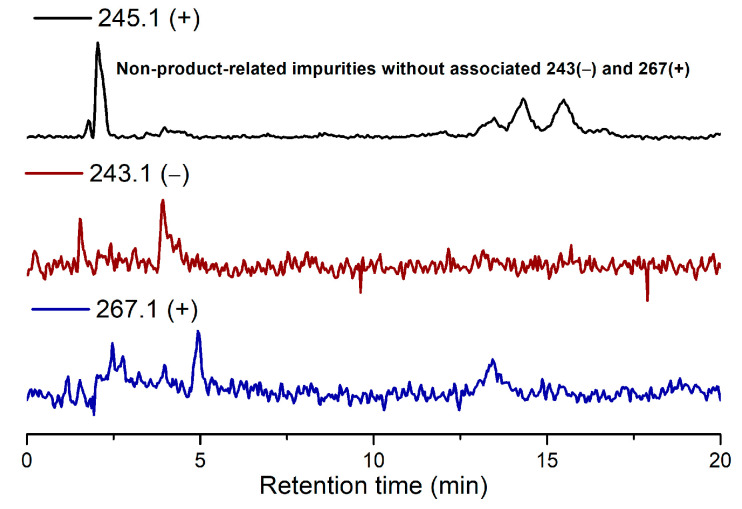
TIC results of the ribosylation of uracil without metal salts and kaolinite.

Due to the low reactivity of uracil, a stoichiometric amount of ribose was used, instead of the one adsorbed on MDC. Multiple metal chlorides (CuCl_2_, FeCl_2_·4H_2_O, MgCl_2_ and CaCl_2_·2H_2_O) and a clay mineral (kaolinite) were added together with the reactants (D-ribose and uracil). Chloride was selected as the anion because of its abundance in the early oceans [20]. The pH of the mixture was adjusted to 5, which should be prebiotically plausible, and the mixture was heated to evaporate water on a shaking dry-bath to simulate the hot–dry conditions on the primordial Earth [21]. The dried sample was added water and analyzed by liquid chromatography—mass spectrometry (LCMS), equipped with a reverse-phase high-performance liquid chromatography (HPLC) column. It was observed that a set of early eluted peaks (1.7, 2.0 and 2.3 min) were strongly associated with the expected masses of 245 ([M + H]^+^), 267 ([M + Na]^+^) and 243 ([M − H]^−^). To confirm the identity of these signals, a co-injection with authentic β-uridine was performed. There were a group of neighboring peaks between 12 min and 16 min, which should be non-product-related impurities, since they did not have all three characteristic masses of 245(+), 267(+) and 243(−). The intensity of the impurity peaks remained unchanged; thus, they were selected as the internal standards. It was found that in the LCMS analysis of the co-injected sample, besides the signal of β-uridine itself, the total ion chromatography (TIC) signal strengths (scanned for 245.1(+)) at 1.7, 2.0 and 2.3 min, also increased sharply, as seen in Figure 2, in the red curve in the upper right of the figure. These observations indicated that the peaks at 1.7, 2.0 and 2.3 min were probably altered forms of β-uridine and/or its isomers. The most probable case was that the products tended to coordinate with metal cations, which caused a polarity increase, and thus an early elution on the reversed-phase HPLC. To further elucidate the effectiveness of using metal ion and clay, a control experiment in the absence of metal salts and kaolinite was conducted, in which the characteristic MS signals and the corresponding LC peaks were not detected (Figure 3). The formation of the products was further determined by HRMS, the analytical method that the Zare lab used for confirming uracil ribosylation. Besides the desired masses of 245.0777 ([M + H]^+^), 267.0587 ([M + Na]^+^) and 243.0622 ([M − H]^−^), it was also interesting to observe the *m*/*z* of the complexes with divalent cations: 134.0265 ([M + Mg]^2+^), 142.0151 ([M + Ca]^2+^), 150.0010 ([M + Fe]^2+^) (see Supplementary Materials).

Although the products cannot be unambiguously determined to be β-uridine itself, these results at least suggested that the product could have the same glycosidic linkage as β-uridine, but in isomeric forms (α-pU, β-pU, α-fU, and β-fU itself). Whether β-furanoside could be formed predominantly was not the primary focus of this study. Instead, we decided to emphasize on the formation of all ribosylated products, and leave the question open on whether the isomers are convertible to β-furanoside under the reaction conditions. The coordination interaction of β-uridine and isomers to metal ions seemed to be strong. We tried to free the nucleosides by treating the sample with cation-exchange resin, but we were unable to detach the metal cations from the products. However, the total yields could be determined by the UV-integral method, no matter whether the chromophore was bound to a cation or in free form. The overall yield of the ribosylation products was between 1.3% and 4.6% on multiple runs, based on the HPLC integration (uncalibrated) of the peaks at 1.7, 2.0 and 2.3 min.

**Figure 4:**
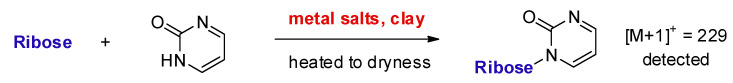
Direct ribosylation of 2-pyrimidinone enabled by metal salts and kaolinite.

Under the same conditions, the ribosylation of cytosine afforded predominantly the 4-ribosylamino products, due to the much greater nucleophilicity of the 4-NH_2_ group that readily reacts with the –CHO group of ribose. A negative mass of 242.1 indicated the formation of the 4-NH_2_-ribosylated cytosine. Under the currently investigated conditions, we have not yet found an effective way to overcome this problem. We have discovered that cavitation (both acoustic and hydrodynamic) is helpful in forming an elevated amount of *N*9 purine nucleoside, compared with 6-ribosyamino products [22], but whether the hypothetical cavitational condition was compatible with the environment rich in metal salts and clay minerals, remains unknown. Therefore, the *N*1-selective ribosylation of cytosine is beyond the scope of this study. To expand the utility of this nucleosidation, we explored the reaction with 2-pyrimidinone, following the same procedure as the ribosylation of uracil (Figure 4).

**Figure 5:**
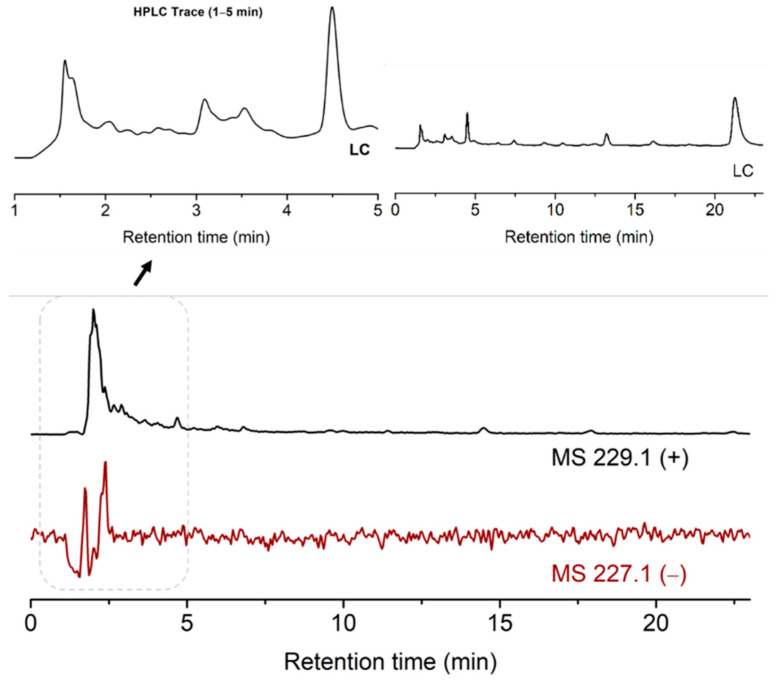
TIC results of the ribosylation of 2-pyrimidinone with metal salts and kaolinite.

To perform this, the pH of the suspension of ribose, 2-pyrimidinone, metal salts and kaolinite was adjusted to 5. The mixture was stirred on an 85 °C heating block for 13 h. The dried mixture was added water and analyzed by LCMS. The desired MS signal of 229 ([M + H]^+^) materialized, indicating the formation of ribosylated 2-pyrimidinone. The MS signal of 227 ([M − H]^−^) was weak, due to the lack of the acidic proton in the ribosylation products. This is different from the case of uracil, which has an acidic and deprotonatable proton at *N*3, thus the negative MS of the products ([M − H]^–^ = 243.1) was much stronger. The product peaks were again in the solvent front, which implied the possible formation of metal complexes (Figure 5).

## 3. Material and Method

### 3.1. Chemicals

D-Ribose (CAS 50-69-1) was purchased from TCI (Shanghai, China) (>98%). Cupric chloride (CAS 7447-39-4) was purchased from Shanghai Xinbao (Shanghai, China) (99%). Iron (II) chloride (CAS 13478-10-9) was purchased from Acmec Biochemical Co., Ltd. (Shanghai, China) (99%). Anhydrous magnesium chloride (CAS 7786-26-2) was purchased from Adamas-Beta (Shanghai, China) (99%). Calcium chloride dehydrate (CAS 10035-04-8) was purchased from General Reagent (Shanghai, China) (AR, 99–103%). Uridine (CAS 58-96-8) was purchased from Adamas-Beta (99%). Uracil (CAS 66-22-8) was purchased from Shanghai Yuanye Bio-Technology Co., Ltd. (Shanghai, China) (98%), and 2-pyrimidinone (CAS 557-01-7) was purchased from Bidepharma Co., Ltd. (Shanghai, China) (97%). Sodium hydroxide (CAS 1310-73-2) was purchased from General Reagent (Shanghai, China) (≥96%). Methanol (CAS 67-56-1) was purchased from Sigma-Aldrich (St. Louis, USA) (HPLC grade, >99.9%). Formic acid (CAS 64-18-6) was purchased from Adamas-Beta (Shanghai, China) (99%).

### 3.2. Instrument

The shaking dry-bath (model# SD1-100) was manufactured by Titan Technology Co., Ltd. (Shanghai, China). Magnetic stirrer (RCT basic) and heating block were manufactured by IKA^®^-Werke GmbH & CO. KG (Janke & Kunkel–Str.10 79219, Staufen, Germany). LCMS analyses were performed on a SHIMADZU LCMS-2020 (1 Nishinokyo Kuwabara-cho, Nakagyo-ku, Kyoto 604-8511, Japan), with a SHIMADZU LC-20AD pump, SPD-20A UV detector, SIL-20AC auto-sampler, and an electrospray ionization (ESI) MS detection mode with a SHARPSIL-U C18 column (S-5 μm, 100 Å, 4.6 mm I.D. × 150 mm), at a detection wavelength of 254 nm, and a flow rate of 1.0 mL/min, with water (pH = 3, adjusted with formic acid, as phase A) and methanol (as phase B) as the eluents. The standard injection volume was 30 μL. The gradient conditions were: 0–3 min, 100% A; 3–300 min, 100% A to 100% B. The elution was stopped at 23 min. The reaction yields were calculated from the integration area of the isomer, divided by the total integration area of the starting material and products. HRMS analyses were performed on a Thermo Fisher Q Exactive Mass Spectrometer (Thermo Fisher Scientific, 168 Third Avenue, Waltham, MA 02451, USA).

### 3.3. Procedure for the Direct Ribosylation of Uracil Enabled by Metal Salts and Kaolinite

Uracil (22.2 mg, 0.20 mmol), D-ribose (450 mg, 3.0 mmol) and multiple metal salts (CuCl_2_ (6.72 mg, 0.05 mmol), FeCl_2_·4H_2_O (9.94 mg, 0.05 mmol), MgCl_2_ (4.76 mg, 0.05 mmol) and CaCl_2_·2H_2_O (7.35 mg, 0.05 mmol)) were added in H_2_O (4 mL, [uracil] = 0.05 M, [D-ribose] = 0.75 M, Σ[multi-metal] = 0.05 M). The solution (1.5 mL) was transferred to a sample vial (2.0 mL) containing kaolinite (120 mg). The pH of the mixture was adjusted to 5 with NaOH. The vial was placed on a shaking dry-bath at 73 °C for 14 h. The dried sample was washed with water (1.5 mL) and filtered. The filtrate was analysed by LCMS and TIC. The elution method was: 0–3 min, 100% buffer A (water/HCOOH buffer, pH = 3), 3–300 min, 100% buffer A to 100% phase B (methanol). The overall yield of the ribosylation products was 1.3–4.6%.

### 3.4. Control Experiment of the Ribosylation of Uracil without Metal Salts and Kaolinite

Uracil (8.33 mg, 0.075 mmol) and D-ribose (168.9 mg, 1.125 mmol) were added in H_2_O (1.5 mL, [uracil] = 0.05 M, [D-ribose] = 0.75 M). The pH of the mixture was adjusted to 5 with hydrochloric acid. The prepared mixture was reacted on a shaking dry-bath at 73 °C for 14 h. The dried sample was washed with H_2_O (1.5 mL) and filtered. The filtrate was analysed by LC-MS and TIC. The elution method was: 0–3 min, 100% buffer A (water/HCOOH buffer, pH = 3), 3–300 min, 100% buffer A to 100% phase B (methanol). No ribosylated uracil was detected.

### 3.5. Procedure for the Direct Ribosylation of 2-Pyrimidinone Enabled by Metal Salts and Kaolinite

In a 2 mL vial, 2-pyrimidinone (9.6 mg, 0.10 mmol), D-ribose (225.2 mg, 1.50 mmol), kaolinite (120 mg), FeCl_2_·4H_2_O (5.0 mg, 0.25 mmol), CuCl_2_·2H_2_O (4.3 mg, 0.25 mmol), MgCl_2_ (2.4 mg, 0.25 mmol) and CaCl_2_·H_2_O (3.7 mg, 0.25 mmol) were added in H_2_O (2 mL, [2-pyrimidinone] = 0.05 M, [D-ribose] = 0.75 M, [FeCl_2_·4H_2_O] = 0.125 M, [CuCl_2_·2H_2_O] = 0.125 M, [MgCl_2_] = 0.125 M, [CaCl_2_·2H_2_O] = 0.125 M). The pH of the solution was adjusted to 5, with aqueous NaOH. The prepared mixture was heated on an 85 °C heating block (IKA^®^) for 13 h. Water (1 mL) was added to the dried mixture. A small part of the reaction mixture (100 μL) was taken and diluted to 1 mL with H_2_O for LCMS analysis. The standard injection volume was 30 μL. The gradient conditions were: 0–3 min, 100% A; 3–300 min, 100% A to 100% B. The elution was stopped at 20 min.

## 4. Conclusions

In conclusion, we have demonstrated an example of the direct coupling of ribose and canonical pyrimidine nucleobase in one step, under prebiotically general conditions. Thus, the previously unachievable direct ribosylation of uracil can be enabled in the presence of metal salts and clay minerals. This transformation is compatible with the metal-doped-clay model proposed by our lab for the RNA World hypothesis. The mechanism for the promoting effect of metal and clay, further optimization of the reaction yield, and the *N*1-selective ribosylation of cytosine are currently under investigation in our lab.

## Data Availability

Data supporting reported results of this study can be found within the main text and the Supplementary Materials of this paper.

## Supplementary Materials

The following are available online at https://www.mdpi.com/article/10.3390/life11121381/s1: HRMS spectra of the product and the TIC traces of the reaction of cytosine.
