# Supplementary material for: A Prebiotic Ribosylation of Pyrimidine Nucleobases Enabled by Metal Cations and Clay Minerals
Source: Life (Basel). 2021 Dec 10;11(12):1381. doi: 10.3390/life11121381 (PMC8707481; doi:10.3390/life11121381)

Supplementary Materials

A Prebiotic Ribosylation of Pyrimidine Nucleobases Enabled by Metal Cations and Clay Minerals

Qian-Qian Chen [1], Ze-Run Zhao [1] and Xiao Wang [1,*]

[1] School of Chemistry and Chemical Engineering, Nanjing University, 163 Xianlin Avenue, Nanjing, 210023, Jiangsu, China.

* Correspondence: wangxiao@nju.edu.cn

[M+H]+ of 245.0777

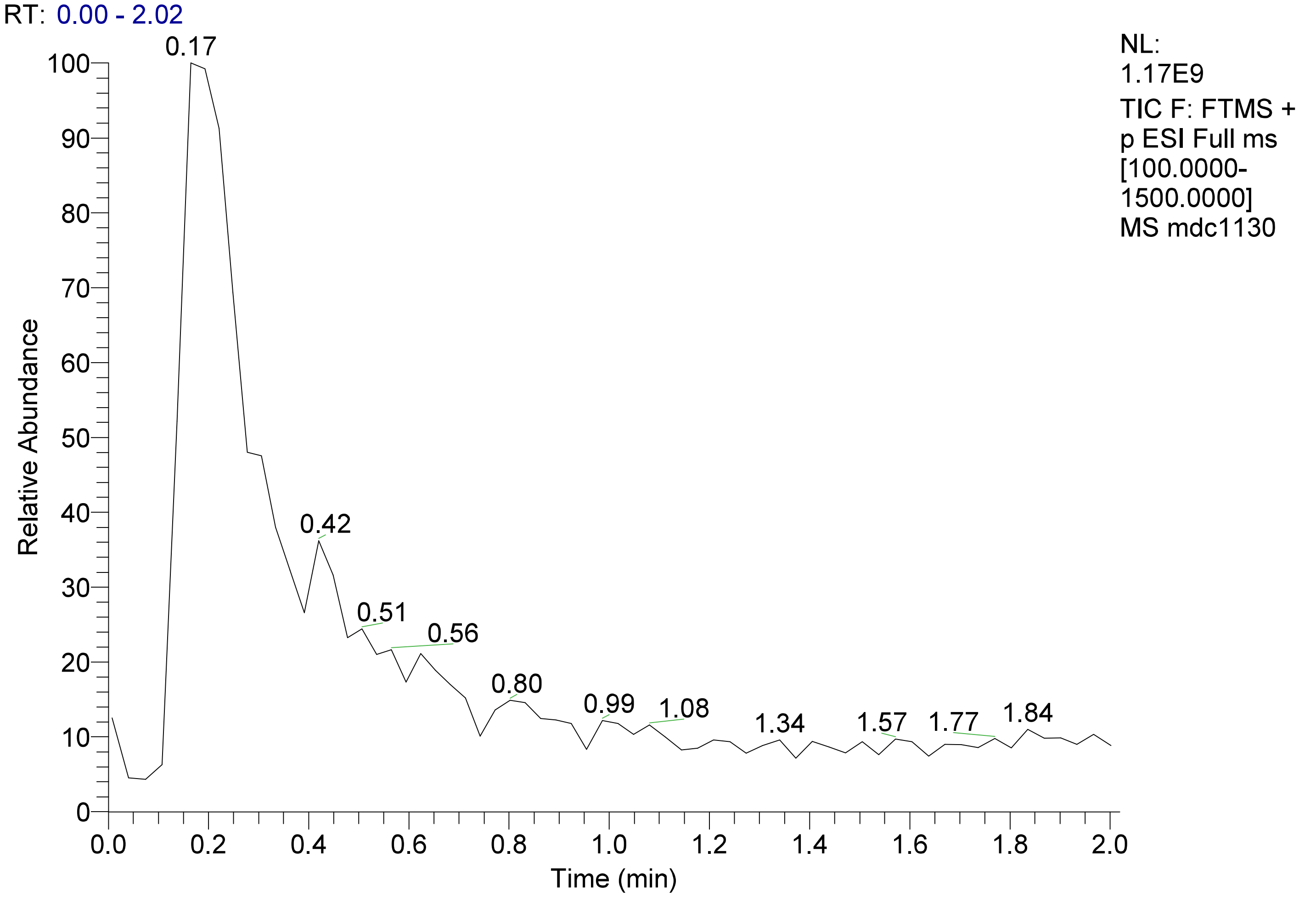

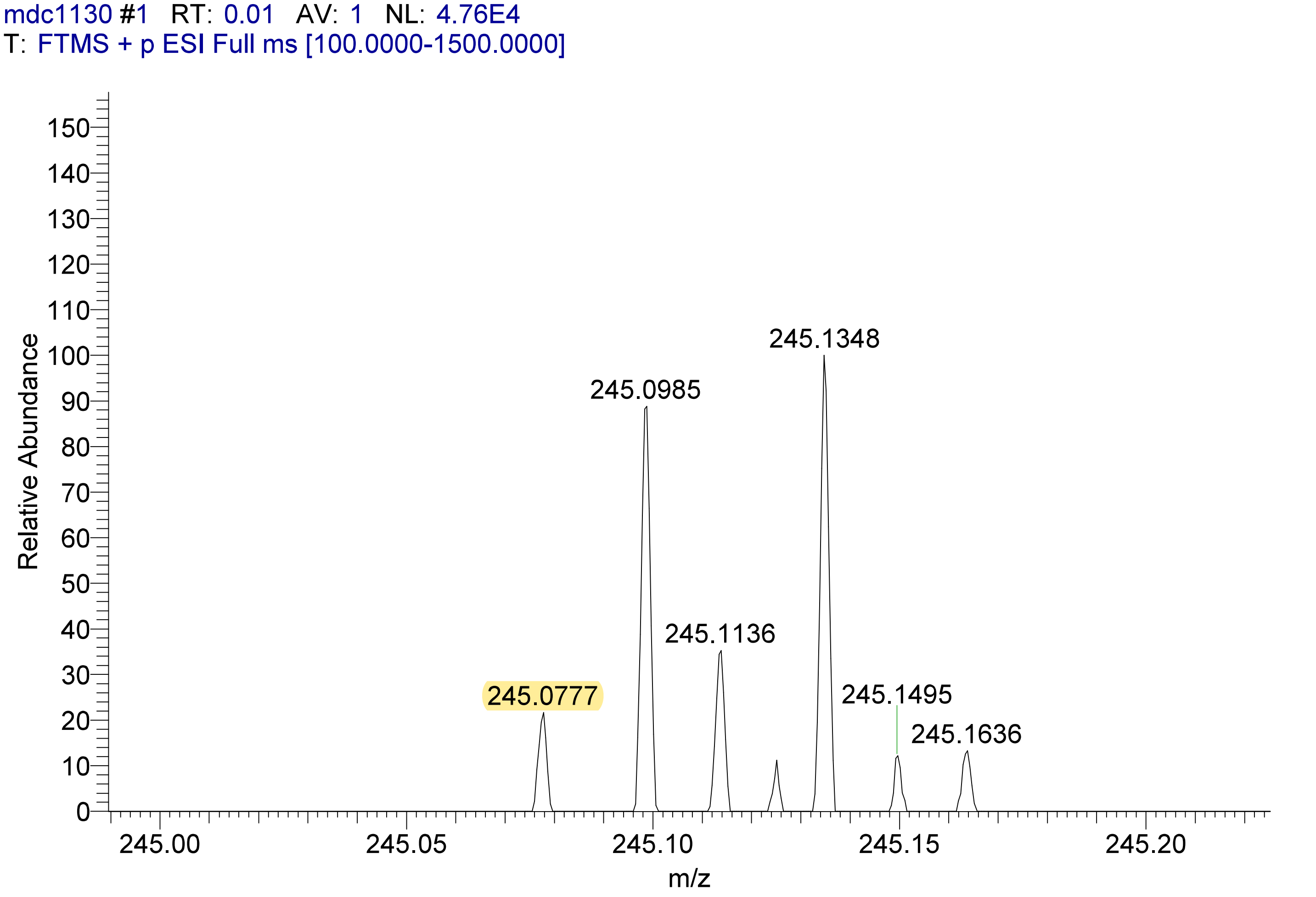

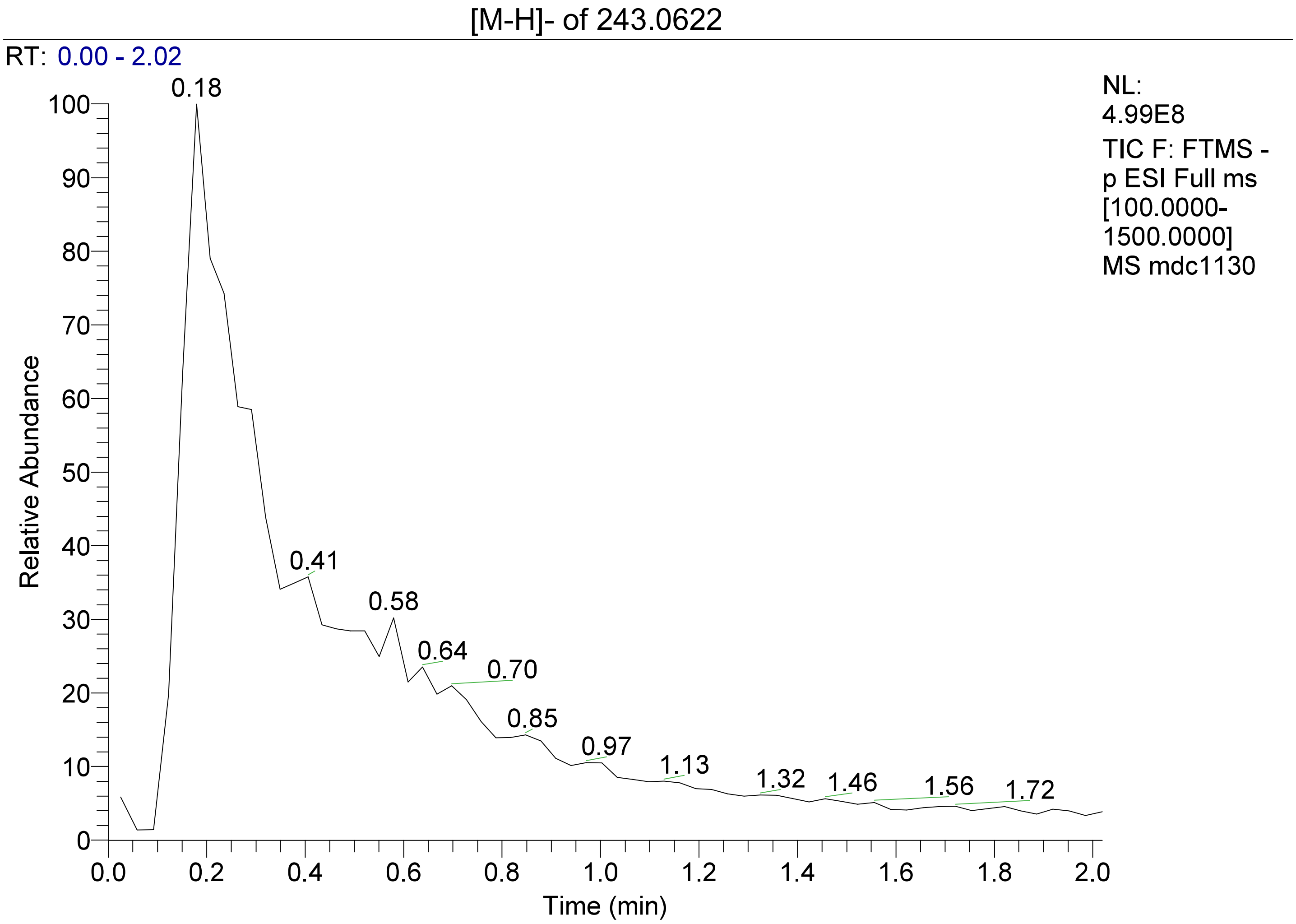
[M-H]- of 243.0622
RT: 0.00 - 2.02
NL:
4.99E8
TIC F: FTMS - p ESI Full ms [100.0000-1500.0000] MS mdc1130
0.18
0.41
0.58
0.64
0.70
0.85
0.97
1.13
1.32
1.46
1.56
1.72
Relative Abundance
Time (min)

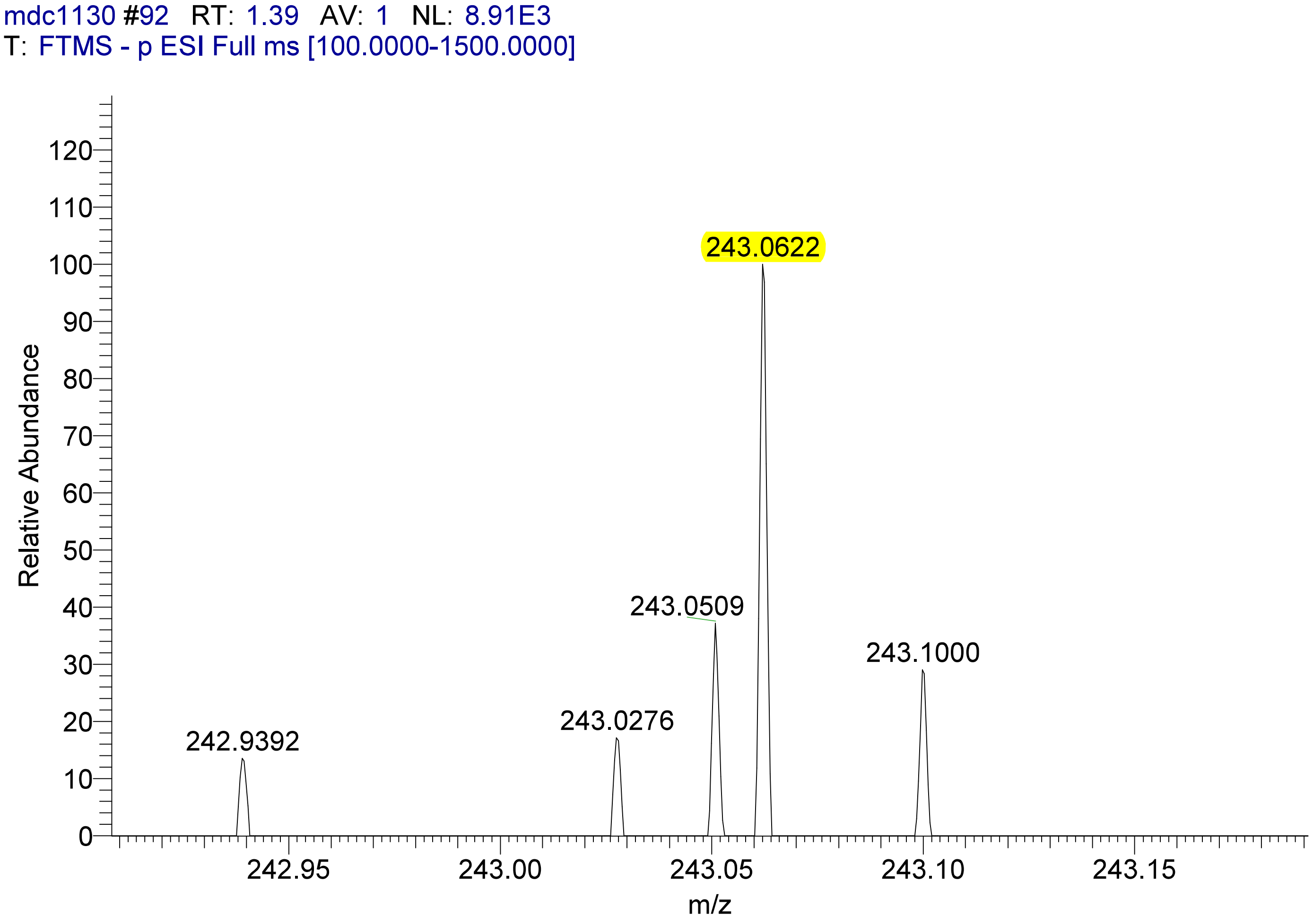
mdc1130 #92 RT: 1.39 AV: 1 NL: 8.91E3
T: FTMS - p ESI Full ms [100.0000-1500.0000]
243.0622
243.0509
243.1000
243.0276
242.9392
Relative Abundance
m/z

[M+Na]+ of 267.0587

RT: 0.00 - 2.02

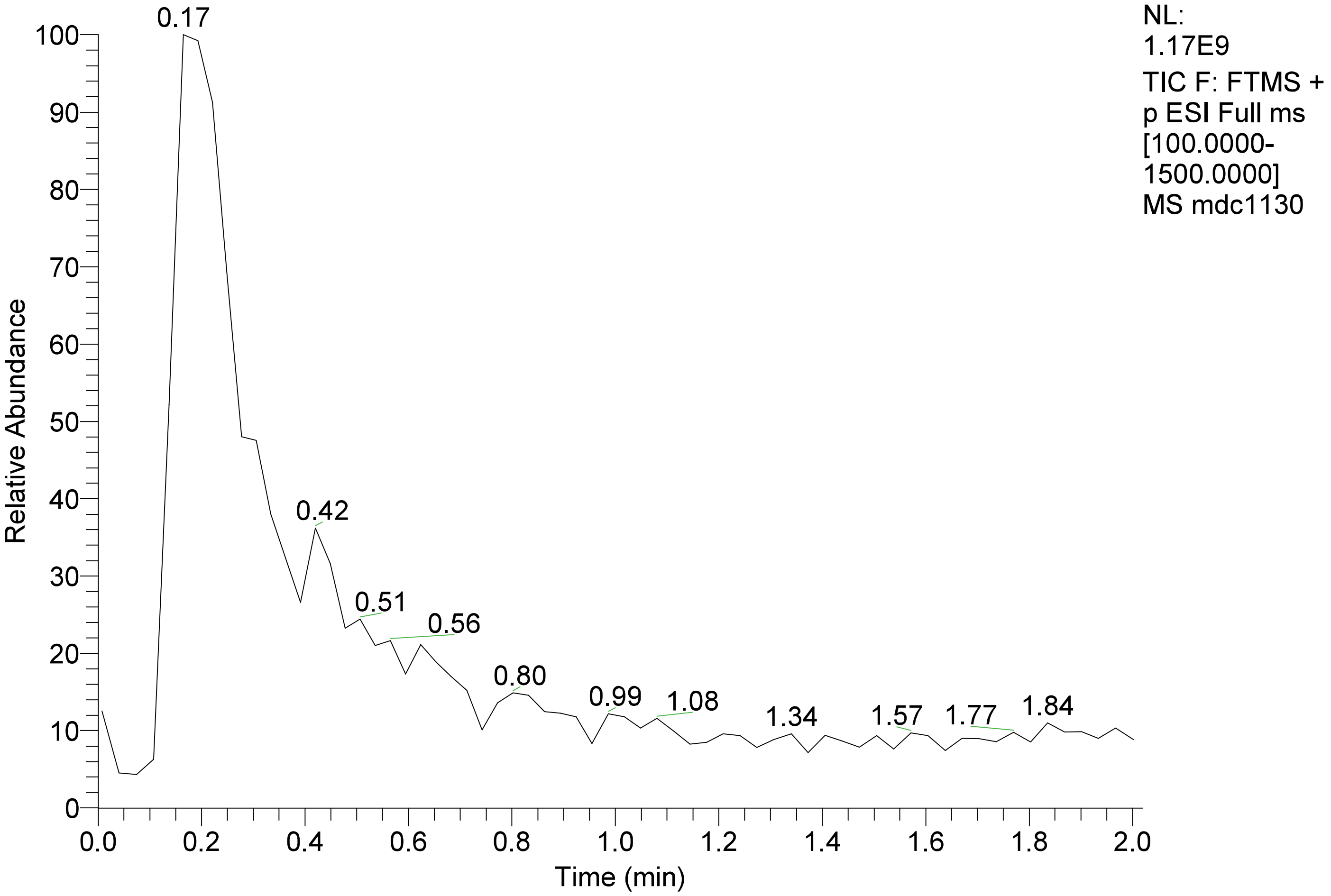

mdc1130 #31 RT: 0.45 AV: 1 NL: 3.56E4
T: FTMS + p ESI Full ms [100.0000-1500.0000]

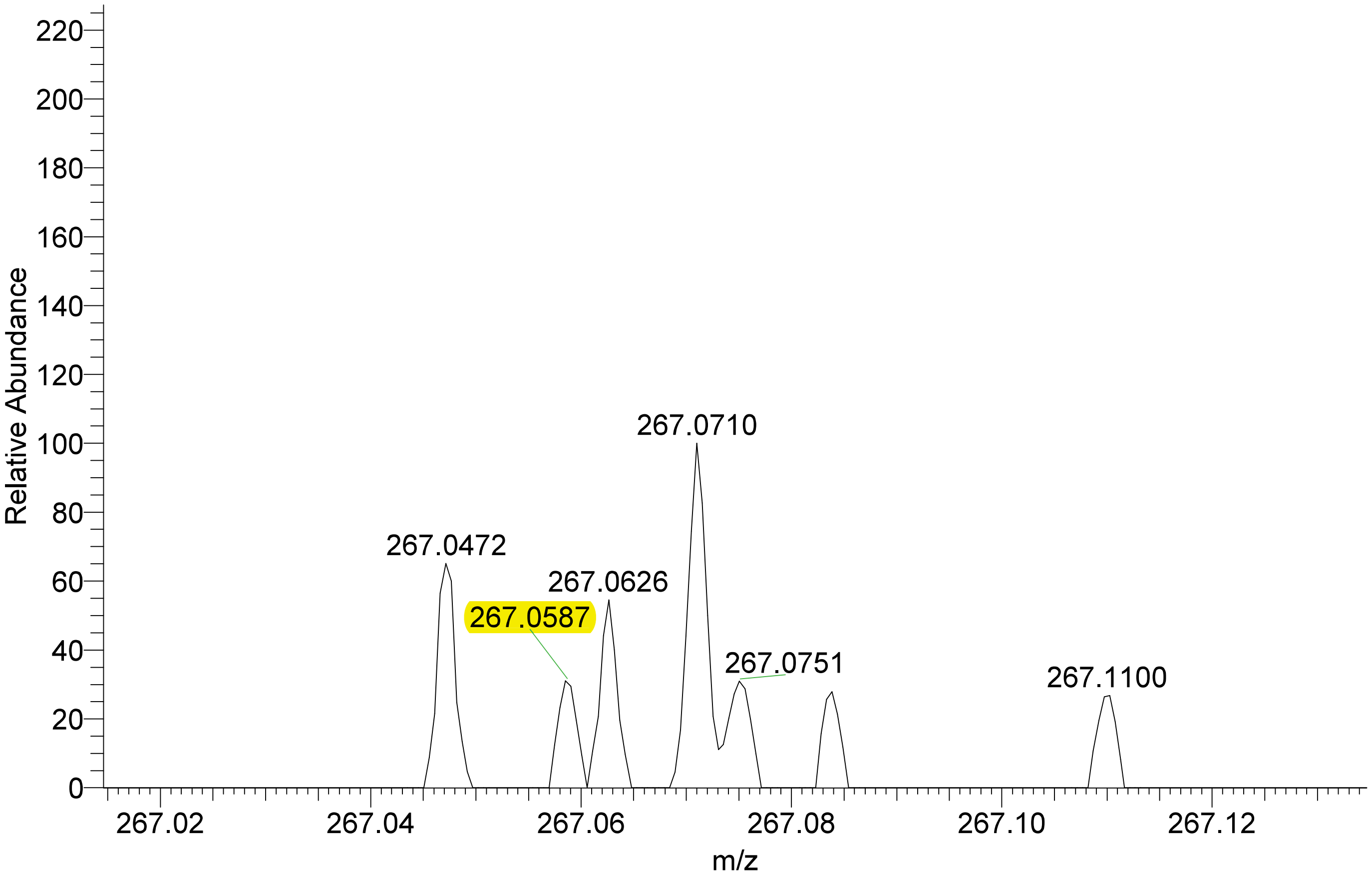

[M+Mg]2+ of 134.0265

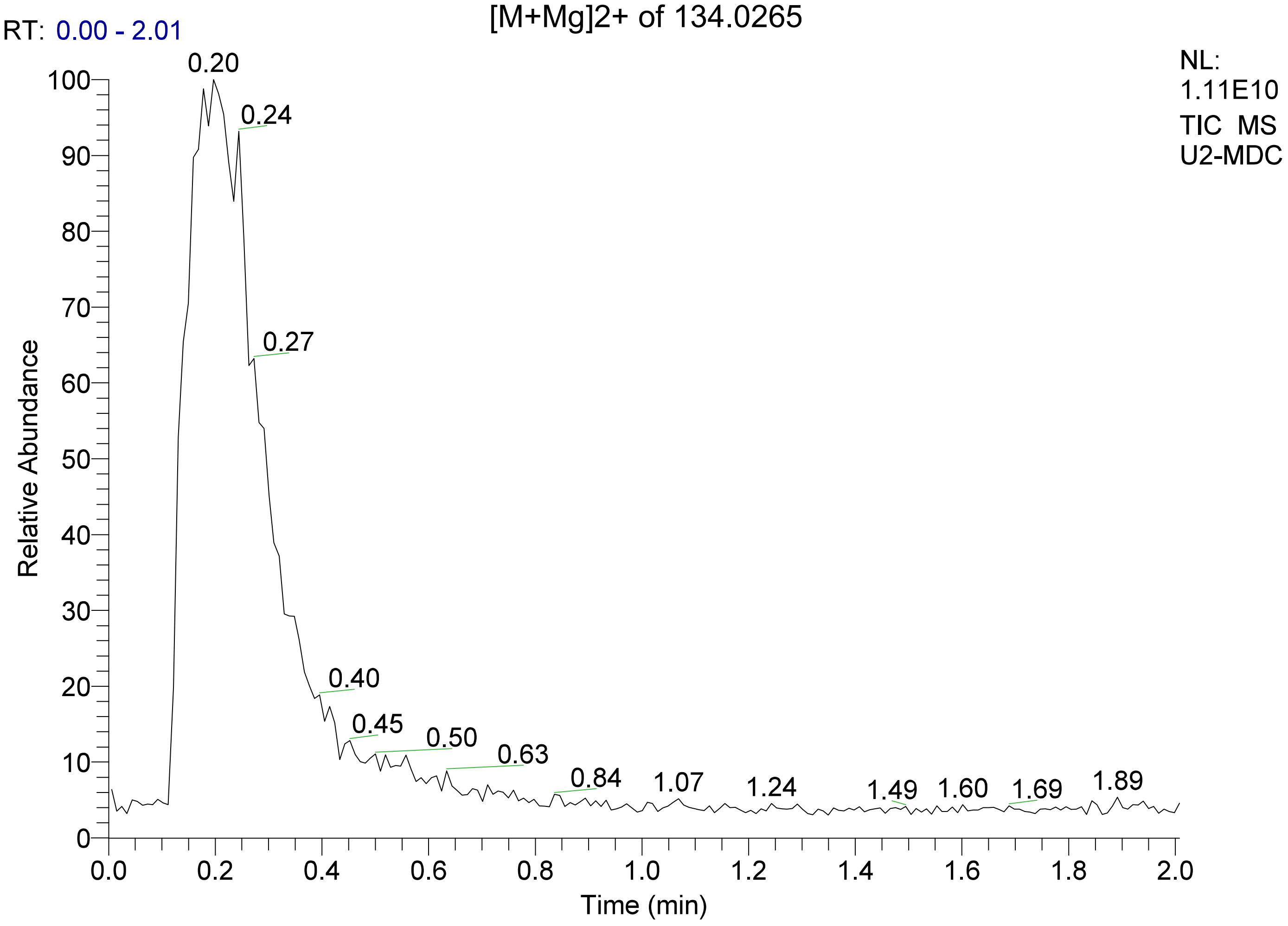

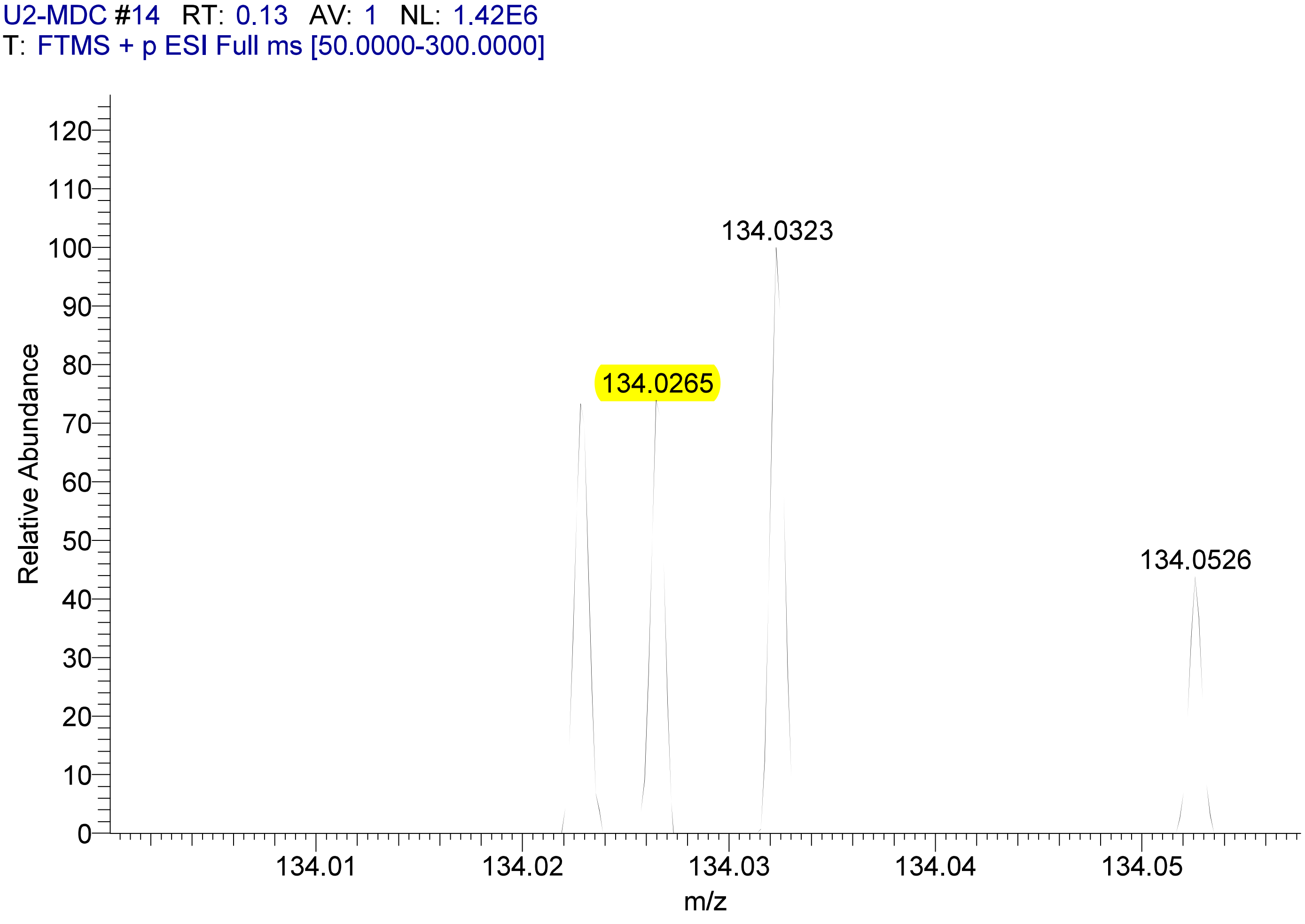

RT: 0.00 - 2.01

[M+Ca]2+ of 142.0151

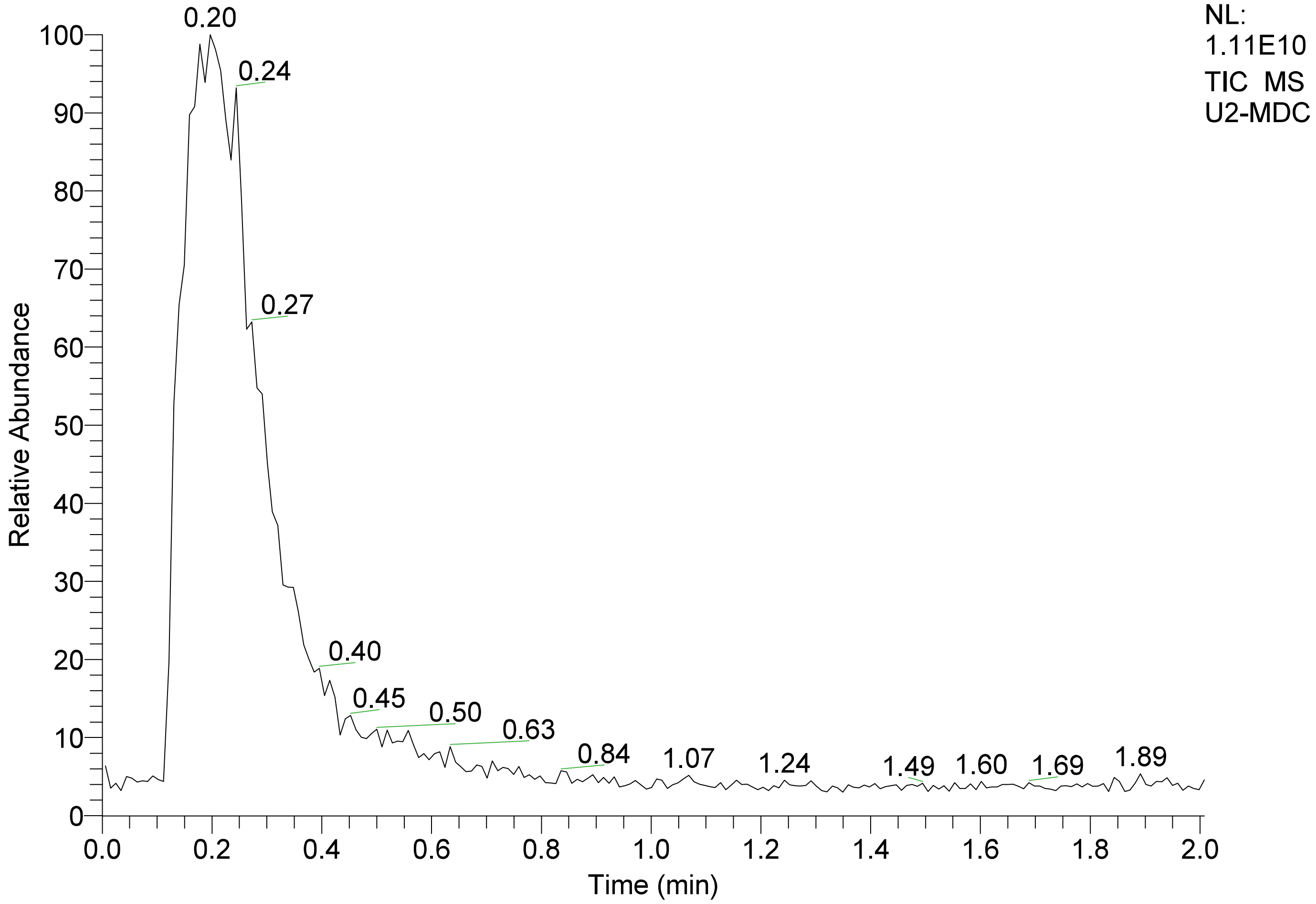

U2-MDC #15 RT: 0.14 AV: 1 NL: 1.94E6
T: FTMS + p ESI Full ms [50.0000-300.0000]

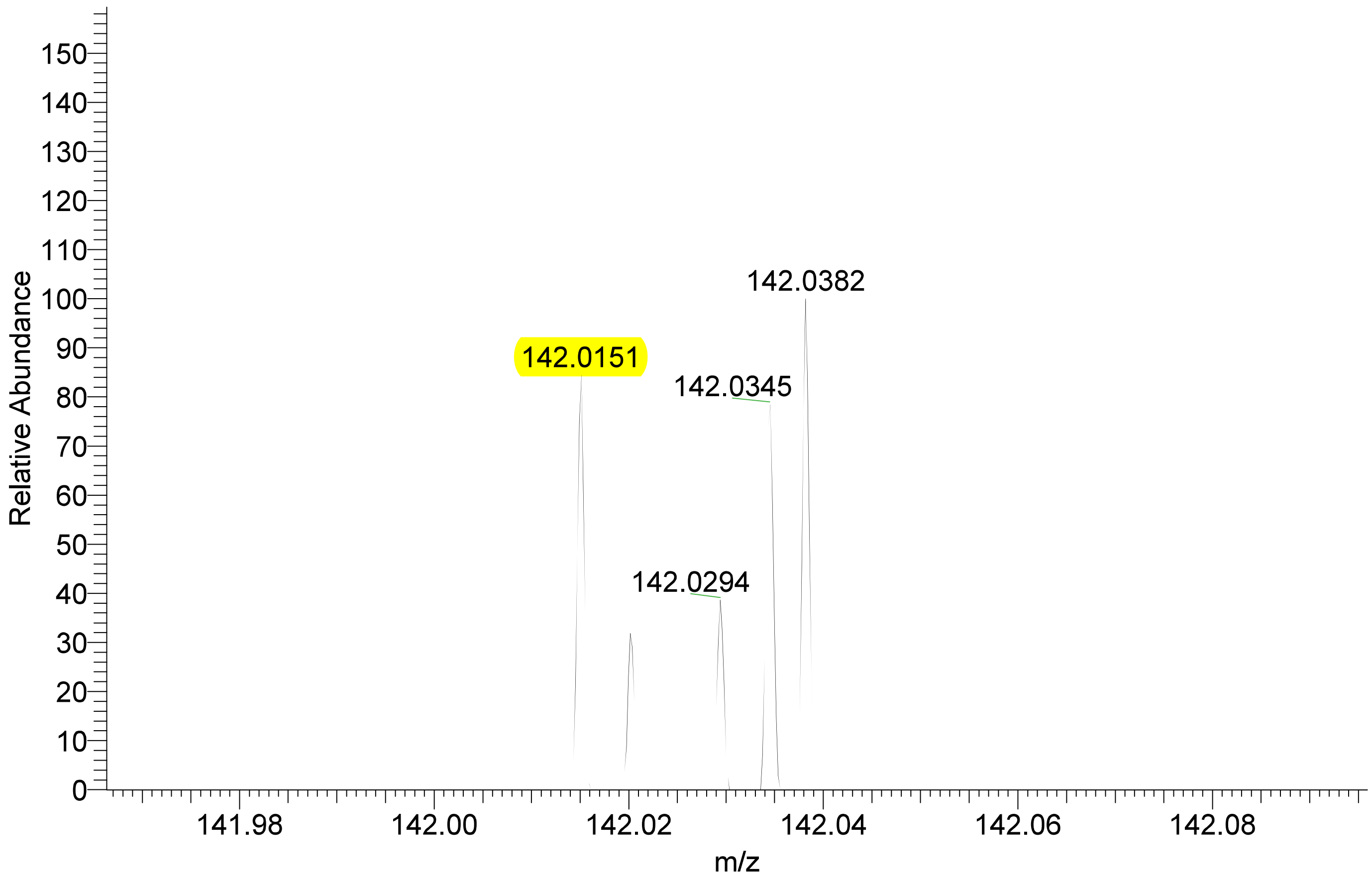

[M+Fe]2+ of 150.0010

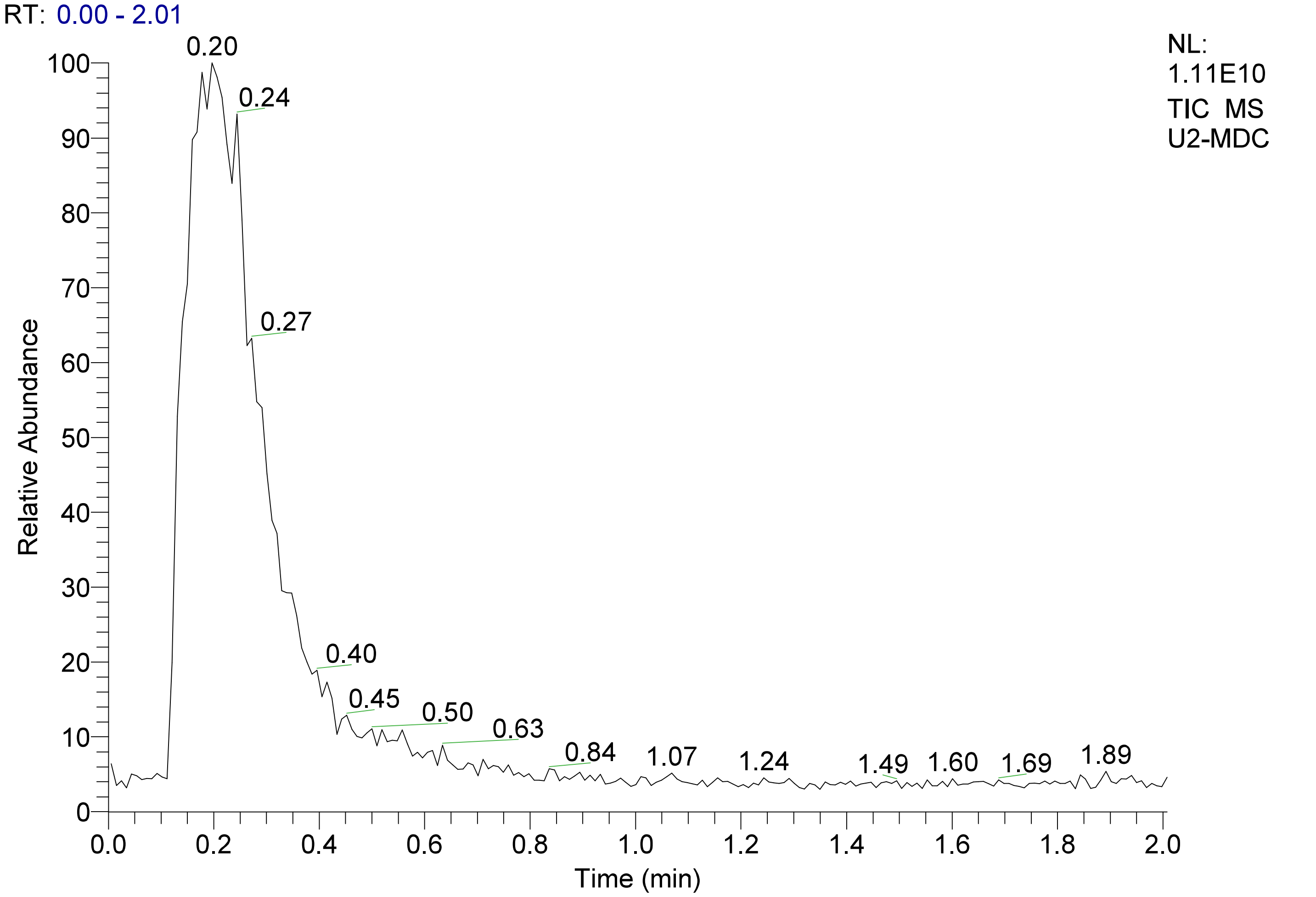

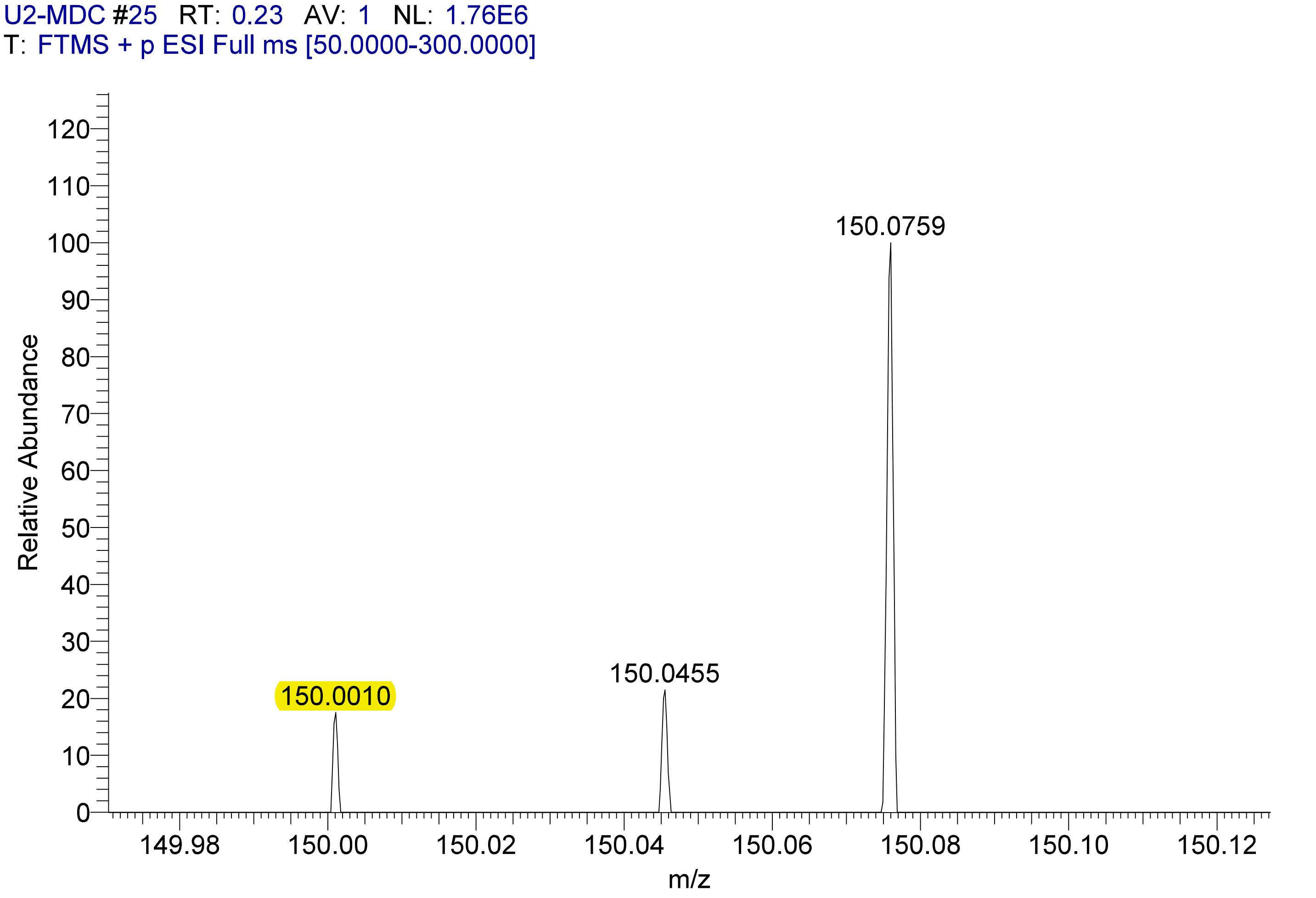

==== Shimadzu LabSolutions Data Report ====

<Chromatogram>

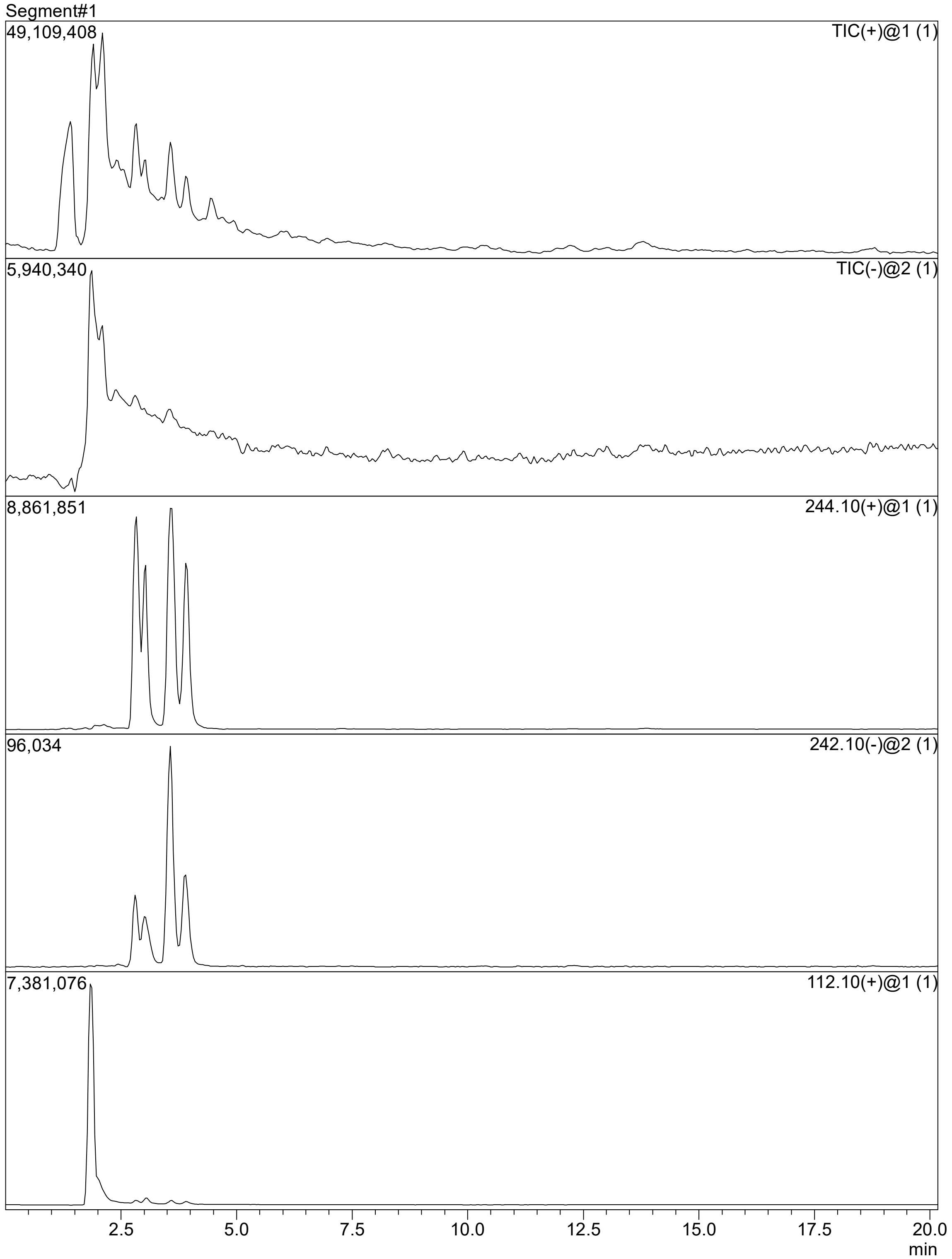

Supplement: Supplementary file 1 [file life-11-01381-s001.zip › Pyrimidine-v2-SM-1.pdf]
